# Chicago Veterinary Society

**Published:** 1897-06

**Authors:** Lawrence Campbell

**Affiliations:** Secretary


Secretary.

### CHICAGO VETERINARY SOCIETY.

A SPECIAL meeting, on April 26, 1897, was called to order by the President, Dr Walker, who stated that the meeting was called for the purpose of deciding whether or not a protest should be made to the Mayor of Chicago against the appointment of a non-graduate and two assistants as veterinarians for the police horses of the city. On motion of Dr. E. L. Quitman, seconded by Dr. Rishel, that a committee of four be appointed by the President to wait on the Mayor and talk the matter over with him, the President appointed Drs. Hughes, Tuthill, Robertson, and P. Quitman. Dr. Walker said he also would attend.

Dr. Tuthill read a very interesting paper on the subject of this appointment; he also touched upon the appointment by the Governor of a non-graduate to the position of State Veterinarian. Moved by Dr. Robertson, seconded by Dr. E. L. Quitman, that the paper read be published in the *Daily Inter-Ocean*. Voted; carried.

On motion, adjourned.

A regular meeting was called to order by the President, May 13, 1897. The roll-call showed twenty-one members present. The Secretary presented a letter from Dr. S. E. Bennett to the Society, requesting that his resignation be accepted, as he had left the State. Motion by Dr. Ryan, seconded by Dr. McGrath, to accept the same. Voted; carried. A bill for stationery being presented by the Secretary for $3.37, motion was made by Dr. McGrath, seconded by Dr. Henderson, that the Treasurer be authorized to pay the same. Voted; carried. On application and examination by the Board of Censors, the two following gentlemen were admitted to full membership: Dr. D. W. McKillip and Dr. O. R. Dubia.

A report was made by Dr. Hughes, chairman of the committee appointed by the President at the last meeting to interview the Mayor in regard to the appointment of a non-graduate to the office of Police Veterinarian. The Doctor stated that the Mayor claimed that he had nothing to do with the appointment, as the office was not the head of a department; and that the Chief of Police was the party to see. The Chief was not seen, as the committee did not feel justified in so doing at the present time. After much discussion it was moved by Dr. E. L. Quitman, seconded by Dr. McGrath, that the Legislative Committee consider the feasibility of drafting a bill to introduce into the Chicago City Council making it a law that the veterinary surgeons employed by the city shall be graduates of recognized veterinary colleges; and that the positions filled by such veterinarians be a separate department from the police and fire departments; and that the legislative committee report at the next meeting their plans for this action. Motion by Dr. E. L. Quitman, seconded by Dr. P. Quitman, to discharge the committee appointed to wait on the Mayor. Voted; carried.

Under new business, Dr. Hughes stated that, owing to the appointment of a non-graduate to the position of State Veterinarian, he intended sending in his resignation as an Assistant State Veterinarian, as he would not consent to work under or assist in any way a non-graduate. This decision was received with applause from the members present. Dr. Ryan followed Dr. Hughes, who also stated that he intended resigning for the same reasons, and he hoped that all the assistants throughout the State would do likewise. A report was made that other assistants not present would also resign. Moved by Dr. E. L. Quitman, seconded by Dr. Robertson, that the Secretary have letters printed which he shall send to all graduate veterinarians in the State, asking those who are now assistant State veterinarians to resign and those who are not to refuse any appointment under the present State Veterinarian or any other non-graduate State Veterinarians, should any appointment be offered to them. That the Secretary give the letters the greatest possible publicity possible in all veterinary journals, farm papers, daily papers, and to the medical profession and public generally, both domestic and foreign. That as the Governor has paid no attention to the requests of the profession in the matter, and has appointed the said non-graduate in direct opposition to the welfare of the profession and the people of the State, when requested privately not to do so, that we now, as a profession, do all we possibly can to redeem the profession from its present condition by interesting the public at large. Moved by Dr. Hughes, seconded by Dr. Ryan, that at the next regular monthly meeting, June 10th, the members of the Society have a banquet. Voted; carried.

It was suggested that the Legislative Committee read a paper at the banquet, divided into sections among the committee, setting forth the proper course of getting a bill through our city council; also as to the meat- and milk-inspection for the city, and how such offices can be placed under the care of veterinarians.

On motion, adjournment.

LAWRENCE CAMPBELL,
Secretary.

